# Green-synthesized zeolite Y-supported zero-valent iron nanocomposite for enhanced adsorptive reduction of hexavalent chromium from aqueous solutions

**DOI:** 10.1039/d5ra09655c

**Published:** 2026-05-15

**Authors:** Nur Fariha Mahmuda, Yanuardi Raharjo, Tokok Adiarto, Handoko Darmokoesoemo, Heru Pramono, Ahmad Fauzi Ismail

**Affiliations:** a Composite Materials & Applications Research Group (MSTRG), Chemistry Department, Faculty of Science and Technology, Universitas Airlangga Surabaya 60115 Indonesia yanuardiraharjo@fst.unair.ac.id; b Chemistry Department, Faculty of Science and Technology Universitas Airlangga Surabaya 60115 Indonesia; c Laboratory of Fisheries Microbiology, Department of Marine Science, Faculty of Fisheries and Marine, Universitas Airlangga Surabaya 60115 Indonesia; d Advances Membrane Technology Research Centre (AMTEC), Universiti Teknologi Malaysia Skudai 81310 Malaysia

## Abstract

Hexavalent chromium (Cr(vi)), a major pollutant in tannery wastewater, poses significant environmental and health risks. Zeolite Y possesses a high cation-exchange capacity but low affinity for Cr(vi), which predominantly exists in anionic forms in aqueous solutions. This study aimed to modify zeolite Y with zero-valent iron nanoparticles (nZVI) to enhance its capacity to adsorb Cr(vi). The zeolite Y/nZVI composite was synthesized using green tea extract. nZVI effectively reduces Cr(vi) to less toxic Cr(iii), which precipitates as Cr(OH)_3_. The composite was characterized using X-ray diffraction (XRD), Fourier-transform infrared (FTIR) spectroscopy, scanning electron microscopy-energy dispersive X-ray spectroscopy (SEM-EDX), particle size analysis (PSA), and pH point-zero charge analysis. The highest Cr(vi) adsorption capacity was achieved under acidic conditions (pH = 1), elevated temperature (343 K), an initial Cr(vi) concentration of 30 ppm, and a contact time of 45 min. The adsorption data were well fitted to the Langmuir isotherm and pseudo-second-order kinetic models. The thermodynamic analysis indicated that the adsorption process was endothermic and spontaneous at higher temperatures. The modification of zeolite Y with zeolite Y/nZVI enhanced the adsorption capacity from 0.9 mg g^−1^ to 7.1 mg g^−1^.

## Abstract 2

Illustration of single-layer formation during the adsorption process.

## Graphical abstract

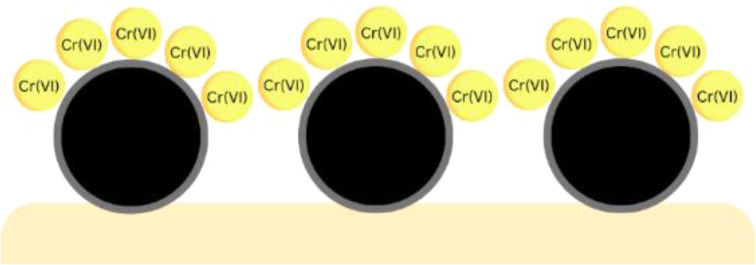

## Introduction

Indonesia's leather industry has grown significantly since the 1970s, with products such as bags, shoes, jackets, and handicrafts being exported to countries such as Hong Kong, Singapore, Thailand, and Japan. Tanning, which is the conversion of raw hides into stable leather, is typically performed using vegetables or chrome. Chrome tanning with Cr(iii) produces soft leather with high thermal and water stability, and thus is more widely adopted than vegetable tanning.^1^ However, only 60–70% of the applied chromium is fixed during tanning, while 30–40% becomes hazardous waste.^2^

Cr (atomic mass 51.9961 g mol^−1^) commonly occurs in two oxidation states: trivalent Cr(iii) and hexavalent Cr(vi). Cr(iii) is relatively stable and exhibits low solubility in water; however, it can be oxidized to Cr(vi) in the presence of oxidizing agents such as MnO_*x*_ and H_2_O_2_, or through photochemical processes.^3^ By contrast, Cr(vi) is highly soluble, more mobile, and significantly more toxic. Its carcinogenic, teratogenic, and mutagenic properties pose serious environmental and human health risks.^4^ The carcinogenic nature of chromium was first recognized in the late 19th century following reports of nasal tumors among workers exposed to chromium compounds.^5^

Among various treatment methods, adsorption has been recognized as an effective technique for Cr(vi) removal from wastewater owing to its high selectivity, efficiency, operational simplicity, and cost-effectiveness.^6^ Adsorbents such as biochar, activated carbon, and zeolites have demonstrated strong potential for eliminating diverse contaminants, including Cr(vi).^7^ Zeolites, crystalline aluminosilicates composed of SiO_4_ and AlO_4_ tetrahedra, can be natural or synthetic. Synthetic zeolites offer tunable pore sizes and surface characteristics as well as superior thermal stability compared with natural zeolites.^8^ Zeolite Y, a faujasite-type zeolite with Si/Al > 1.5, possesses a high ion-exchange capacity and has been widely employed for heavy metal removal from wastewater.^9,10^

Nevertheless, Cr(vi) generally exists as anions (HCrO_4_^−^, Cr_2_O_7_^2−^, CrO_4_^2−^) depending on pH, resulting in low affinity with the Na–Y zeolite.^10–12^ Therefore, the modification of zeolite Y is necessary to enhance its adsorption capacity for chromium anions. To address this challenge, recent studies have explored the immobilization of zero-valent iron nanoparticles (nZVI) on zeolite surfaces. nZVI reduces Cr(vi) to Cr(iii), which subsequently precipitates as Cr(OH)_3_, while the zeolite matrix prevents nZVI agglomeration.^13,14^ Owing to its large surface area, low cost, low toxicity, and strong reductive capability, nZVI has been widely applied for the remediation of organic and inorganic pollutants.^15^

Conventional nZVI synthesis methods, such as top-down milling or bottom-up chemical reduction with NaBH_4_, face limitations including high cost, toxicity, and energy consumption, as well as hydrogen gas generation.^15^ To overcome these drawbacks, green synthesis approaches using plant extracts as reducing and stabilizing agents have been developed. Extracts of *Eucalyptus*, *Verbascum thapus*, green tea, and black tea have been successfully used to produce nZVI.^16–18^ In particular, green tea extract, which is rich in polyphenols, functions both as a reducing agent and stabilizer, improving reactivity and minimizing nanoparticle aggregation.^19^

Beyond its synthesis efficiency, the use of green tea extract offers distinct sustainability advantages compared with conventional NaBH_4_-based reduction. As renewable and biodegradable resources, plant-derived polyphenols reduce the reliance on hazardous synthetic chemicals and limit the formation of environmentally persistent byproducts.^20^ By contrast, NaBH_4_-based synthesis raises safety and environmental concerns associated with its high reactivity and the generation of boron-containing residues.^21^ Additionally, green synthesis is typically conducted in aqueous media under mild reaction conditions, in line with green chemistry principles that emphasize renewable feedstocks and environmentally responsible processes. Thus, this approach offers a sustainable route for the preparation of nZVI while preserving its functional performance.

Based on this concept, zeolite Y/nZVI composites were synthesized using green tea extract as a reducing agent to enhance Cr(vi) adsorption. The effects of pH and temperature on adsorption performance were evaluated, while structural and surface properties were characterized using X-ray diffraction (XRD), Fourier-transform infrared (FTIR) spectroscopy, scanning electron microscopy-energy-dispersive X-ray spectroscopy (SEM-EDX), particle size analysis (PSA), and pH point-zero charge (pH_pzc_) analysis.

## Experimental

### Materials

Commercial dried green tea leaves were purchased from a local market (Surabaya, Indonesia). All the chemicals used in this study were of analytical grade. To synthesize the zeolite Y/nZVI materials, iron (iii) chloride hexahydrate (FeCl_3_·6H_2_O) (Merck), ethanol absolute (Supelco), and deionized (DI) water were used. For the adsorption experiment, potassium dichromate (Merck), sodium hydroxide (NaOH) (Supelco), 98% sulfuric acid (H_2_SO_4_) (Merck), hydrochloric acid (HCl) (Merck), and 1,5-diphenylcarbazide (Sigma-Aldrich) were used.

### Procedure

#### Green tea extraction

Polyphenol extraction from dried green tea was based on the methods of Soliemanzadeh and Fekri.^22^ The commercial leaves of green tea were used as sources of polyphenols. Dried green tea (20 g) was ground and added to 200 mL of DI water. The solution was heated at 80 °C for 60 min and then filtered through a glass microfiber filter to obtain the final extract.

#### Green synthesis of nZVI and zeolite Y/nZVI

Zeolite Y/nZVI was synthesized by modifying the method of Soliemanzadeh and Fekri.^22^ Zeolite Y (4 g) was added to 50 mL of 0.1 M FeCl_3_·6H_2_O solution in a 250 mL Erlenmeyer flask (8% w/v) and magnetically stirred for 30 min. Then, 50 mL of the green tea extract was added dropwise to the zeolite Y mixture and continuously stirred for 30 min. The black suspension was centrifuged at 4000 rpm for 15 min to collect the residue. The residue was washed twice with DI water and twice with absolute ethanol. The black residue was then transferred to an evaporating dish and dried in a vacuum desiccator at room temperature (34 °C). Finally, the powdered zeolite Y/nZVI was stored in a vacuum desiccator until further use to prevent oxidation. nZVI was synthesized using the same method, but without adding zeolite Y.

### Characterization

#### XRD

Zeolite Y/nZVI and zeolite Y were characterized by XRD to verify their synthesis. The XRD spectra were measured using a Rigaku Ultima diffractometer in the 2*θ* range of 5–50°.

#### FTIR spectroscopy

FTIR spectra were acquired using a PerkinElmer spectrometer in the range of 4000–400 cm^−1^ to analyze the functional groups of zeolite Y, zeolite Y/nZVI, and zeolite Y/nZVI-Cr.

#### SEM-EDX

An SEM-EDX spectrometer (Hitachi) was used to analyze the morphologies and elemental compositions of zeolite Y and zeolite Y/nZVI before and after adsorption. The samples were sputter-coated with a gold layer containing polyphenolic powder before analysis.

#### PSA

A particle size analyzer was used to measure the nanoparticle size of nZVI.

#### pH_pzc_

The pH_pzc_ of zeolite Y/nZVI was measured as described by Budiana *et al.*^23^ The pH_pzc_ of zeolite Y/nZVI represents the pH at which the adsorbent is neutrally charged. The initial pH of 0.01 M NaCl (10 mL) was adjusted between 1 and 9 by adding 0.1 M NaOH or 0.1 M HCl and monitored using a pH meter. Each pH-adjusted solution was placed in 50 mL Erlenmeyer flask. Subsequently, zeolite Y/nZVI (30 mg) was added to each Erlenmeyer flask and shaken using an orbital shaker for 24 h at room temperature. The final pH value was measured using a pH meter. The curve of ΔpH *vs.* initial pH was plotted to determine the pH_pzc_.

### Batch adsorption experiment

#### Effect of pH

To determine the optimal pH, it was varied among 1, 2, 3, 5, 7, and 9 by adding 0.1 M HCl or 0.1 M NaOH. Subsequently, 1 mL of 1000 ppm Cr(vi) solution was transferred into six 50 mL volumetric flasks. Each solution was then diluted with pre-adjusted distilled water to obtain 20 ppm Cr(vi) solutions with pH values of 1, 2, 3, 5, 7, and 9. Then, 25 mL of the 20 ppm Cr(vi) solution was transferred to a 100 mL beaker, and 0.05 g of zeolite Y/nZVI was added. The adsorption experiments were conducted in batch mode at room temperature for 30 min. The residual Cr(vi) concentration was analyzed using a UV-vis spectrophotometer at a wavelength of 540 nm. The adsorption capacity (*q*_ads_) was calculated using eqn (1).1
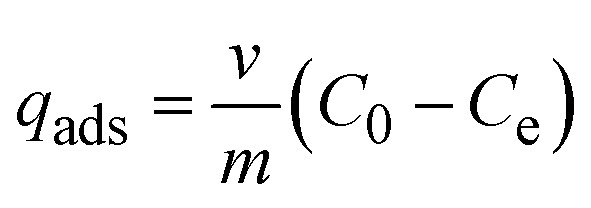
where *C*_0_ is the initial concentration (mg L^−1^), *C*_e_ is the equilibrium concentration (mg L^−1^), *V* is the feed solution volume, and *m* is the mass of the adsorbent.

#### Effect of temperature

Temperature optimization was conducted at 303, 313, 323, 333, and 343 K. Zeolite Y/nZVI (0.05 g) was added to 25 mL of a 20 ppm Cr(vi) solution at pH 1. The mixture was then shaken at room temperature for 30 min.

To determine the spontaneity of the adsorption process, the mathematical expressions of the Gibbs free energy (Δ*G*°) are shown in eqn (2)–(4).

2 Δ*G*° =−*RT* ln *K*_a_

3 Δ*G*° = Δ*H*° − *T*Δ*S*°

4
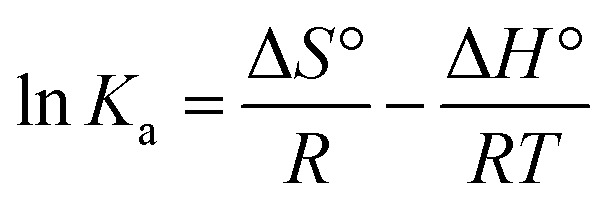
where *R* is the universal gas constant (J mol^−1^ K^−1^), *T* is the reaction temperature (*K*), and *K*_a_ is the thermodynamic equilibrium constant obtained from *q*_e_/*C*_e_. The Δ*H*° value can be determined from the slope of the linear regression plot of ln *K*_a_*vs.* 1/*T*.^1^

#### Effect of initial concentration

To optimize the initial concentration, it was varied among 10, 20, 30, 40, and 50 ppm. Zeolite Y/nZVI (0.05 g) was added to 25 mL of Cr(vi) solution at the specified concentrations at pH 1. The mixture was then shaken at room temperature for 30 min.

To determine the adsorption mechanism, Langmuir and Freundlich models were used, as expressed in eqn (5) and (6), respectively.5
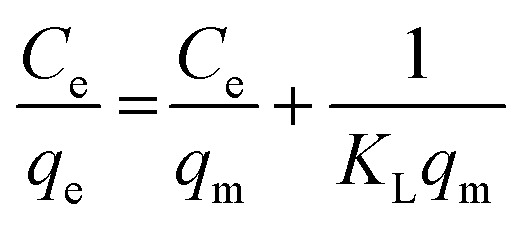
6
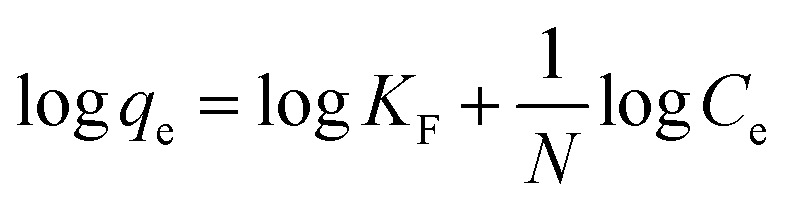
where *q*_e_ is the adsorption capacity at equilibrium (mg g^−1^), *C*_e_ is the equilibrium concentration of the analyte in the solution (mg L^−1^), *q*_m_ is the maximum adsorption capacity (mg g^−1^), and *K*_L_ is the Langmuir constant (L mg^−1^). *K*_F_ (L mg^−1^) and *N* are the Freundlich coefficients related to the adsorption capacity and adsorption intensity, respectively, both of which are temperature-dependent.

#### Effect of contact time

The contact times were 5, 10, 15, 30, 45, 60, and 90 min. Zeolite Y/nZVI (0.05 g) was added to 25 mL of a 30 ppm Cr(vi) solution at pH 1. The adsorption experiments were conducted at room temperature for varying durations.

The adsorption process was analyzed by investigating the adsorption kinetics. The adsorption kinetics were studied using pseudo-first-order, pseudo-second-order, and intra-particle diffusion models. Eqn (7)–(9) are the linear equations of the three models.

7 ln(*q*_e_ − *q*_*t*_) = ln *q*_e_ − *k*_1_*t*

8
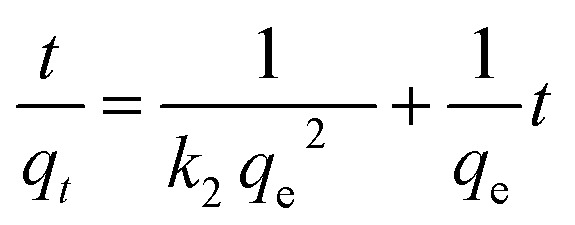

9 *q*_*t*_ = *k*_p_*t*^1/2^ + *C*

where *q*_e_ and *q*_*t*_ represent the Cr(vi) adsorbed (mg g^−1^) at equilibrium and time *t* (min), respectively; *k*_1_ (min^−1^) and *k*_2_ (min^−1^) are the rate constants of the pseudo-first-order and pseudo-second-order models, respectively; and *k*_p_ (mg g^−1^ min^−1/2^) is the intra-particle diffusion rate constant.^24^

## Results and discussion

### Characterization of zeolite Y/nZVI

#### XRD

**Fig. 1:**
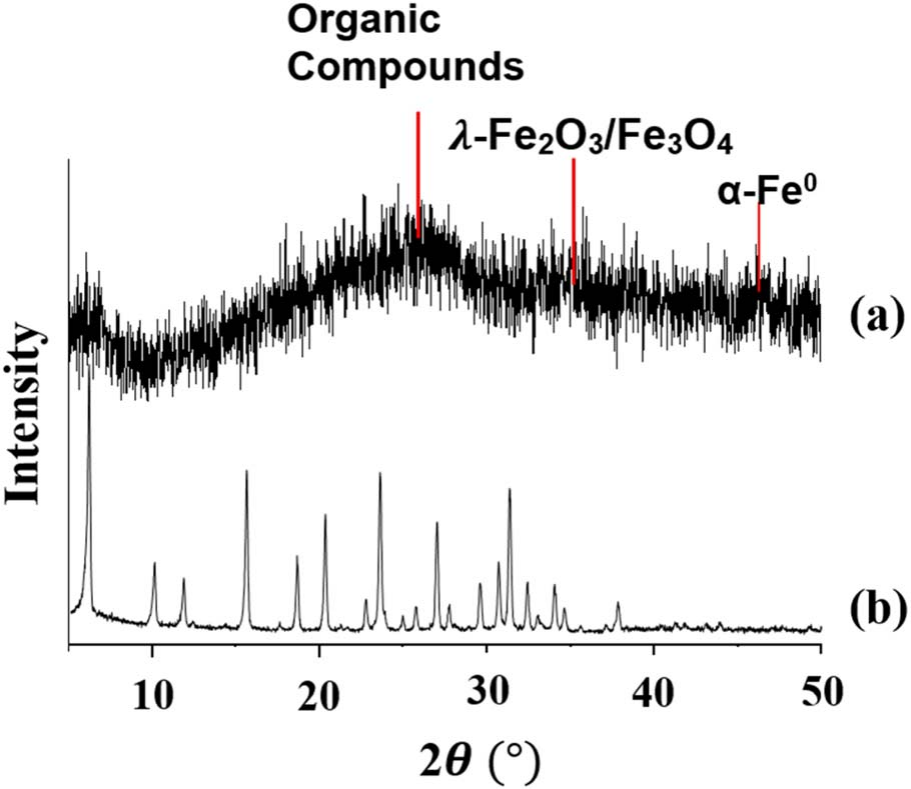
XRD spectra of (a) zeolite Y/nZVI and (b) zeolite Y.

The XRD patterns of the zeolite Y (Fig. 1b) show sharp peaks at 2*θ* = 6.13°, 11.79°;15.56°;23.57° and 31.27°. These peaks correspond to those of the zeolite Y standard reported by Treacy and Higgins (6.19°;11.86°, 15.61°, 23.58 and 31.31°).^25^

The XRD profiles of zeolite Y/nZVI (Fig. 1a) exhibit a broad shoulder peak at 2*θ* = 25°, corresponding to polyphenols and caffeine in green tea extract, which is in accordance with the report of Xiao *et al.*^19^ These organic compounds play an important role as capping agents to prevent Fe^0^ oxidation. The weak peak at 2*θ* = 44.9° indicates amorphous Fe^0^ nanoparticles (nZVI),^19^ which can be attributed to the chelation of iron species by tea polyphenols during synthesis.^26^ This amorphous structure has also been observed in the synthesis of Fe^0^ nanoparticles using plant extracts such as *Eucalyptus*, *Eichhornia crassipes* leaves, *Nephrolepis auriculata*, and *Psidium guajava* leaves.^20,27–29^ In addition, the peak at 2*θ* = 35.7°is attributed to *λ*-Fe_2_O_3_/Fe_3_O_4_, indicating the formation of an iron oxide layer surrounding the Fe^0^ core. This supports the core–shell structure of the synthesized zeolite Y/nZVI composite, which is consistent with previous reports.^19^

##### FTIR spectroscopy

**Fig. 2:**
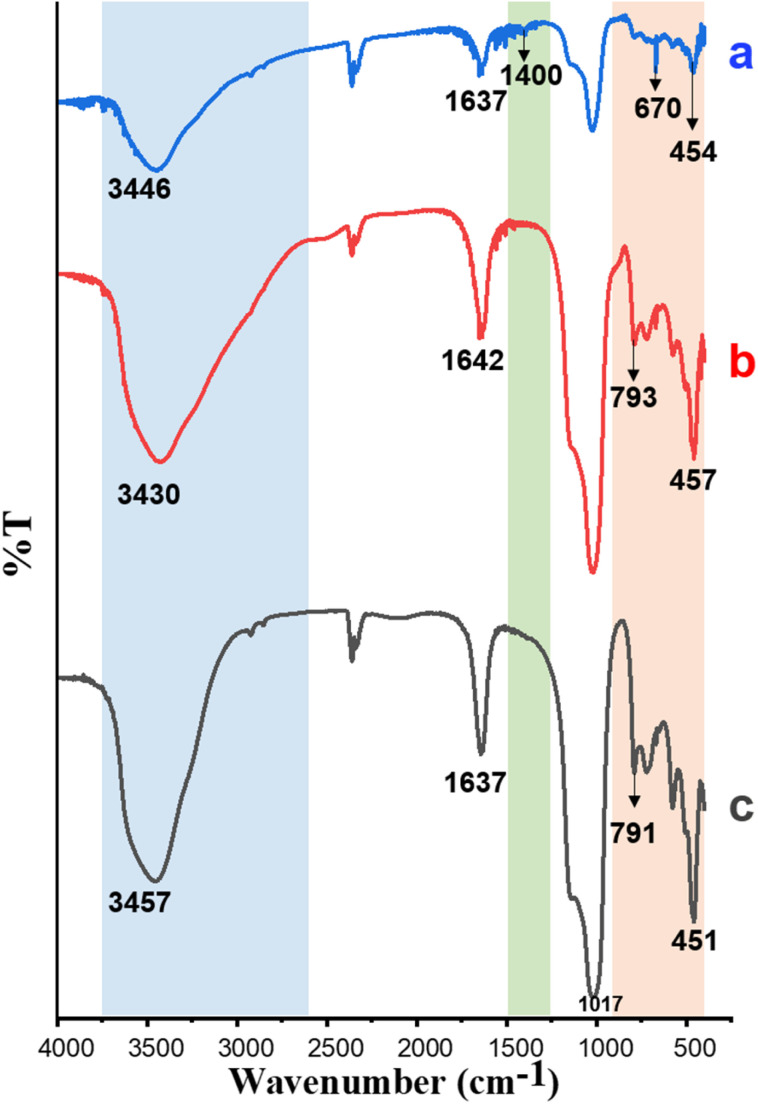
FTIR spectra of (a) zeolite Y/nZVI-Cr, (b) zeolite Y/nZVI, and (c) zeolite Y.

The FTIR spectra of zeolite Y, Y/nZVI, and Y/nZVI-Cr(vi) are shown in Fig. 2. In the spectrum of zeolite Y (Fig. 2c), the band at 3457 cm^−1^ is assigned to the free hydroxyl vibration of the silanol groups. The peaks at 1637–1642 cm^−1^ correspond to the bending of O–H in adsorbed water molecules.^30^ The absorption peaks at 1017, 791, and 451 cm^−1^ are due to the stretching and bending vibrations of S–O–Si, Al–O–Si, and AlAlOH bonds in zeolite Y, which are replaced by weaker Si–O–Fe, AlFeOH, and FeOOH bonds after being modified to zeolite Y/nZVI (Fig. 2b).^30,31^ The bands at 3200–3450 cm^−1^ in zeolite Y/nZVI and zeolite Y/nZVI-Cr indicate the presence of OH groups bound to Fe^0^ or iron oxyhydroxide.^32^ The broad band at 3430 cm^−1^ and narrow band at 1642 cm^−1^ in zeolite Y/nZVI can be attributed to the O–H stretching vibrations of hydroxyl groups and C=C stretching vibrations of aromatic structures associated with residual organic compounds such as polyphenols from the green tea extract, respectively.^16,19,32^

After Cr(vi) adsorption (Fig. 2c), the intensity of the band at <900 cm^−1^ decreases owing to Fe^0^ oxidation on the surface of zeolite Y/nZVI as a result of Cr(vi) reduction.^32^ Meanwhile, the weak band at 1400 cm^−1^ in the spectrum of zeolite Y/nZVI-Cr is assigned to the Fe–OH bending vibration resulting from the surface oxidation of the Fe^0^ core, forming a shell consisting of iron oxides and oxyhydroxides such as magnetite (Fe_3_O_4_), hematite (Fe_2_O_3_), and lepidocrocite (*γ*-FeOOH).^32^ The band at 670 cm^−1^ is attributed to the Fe–O vibration.^33,34^

##### SEM-EDX

**Fig. 3:**
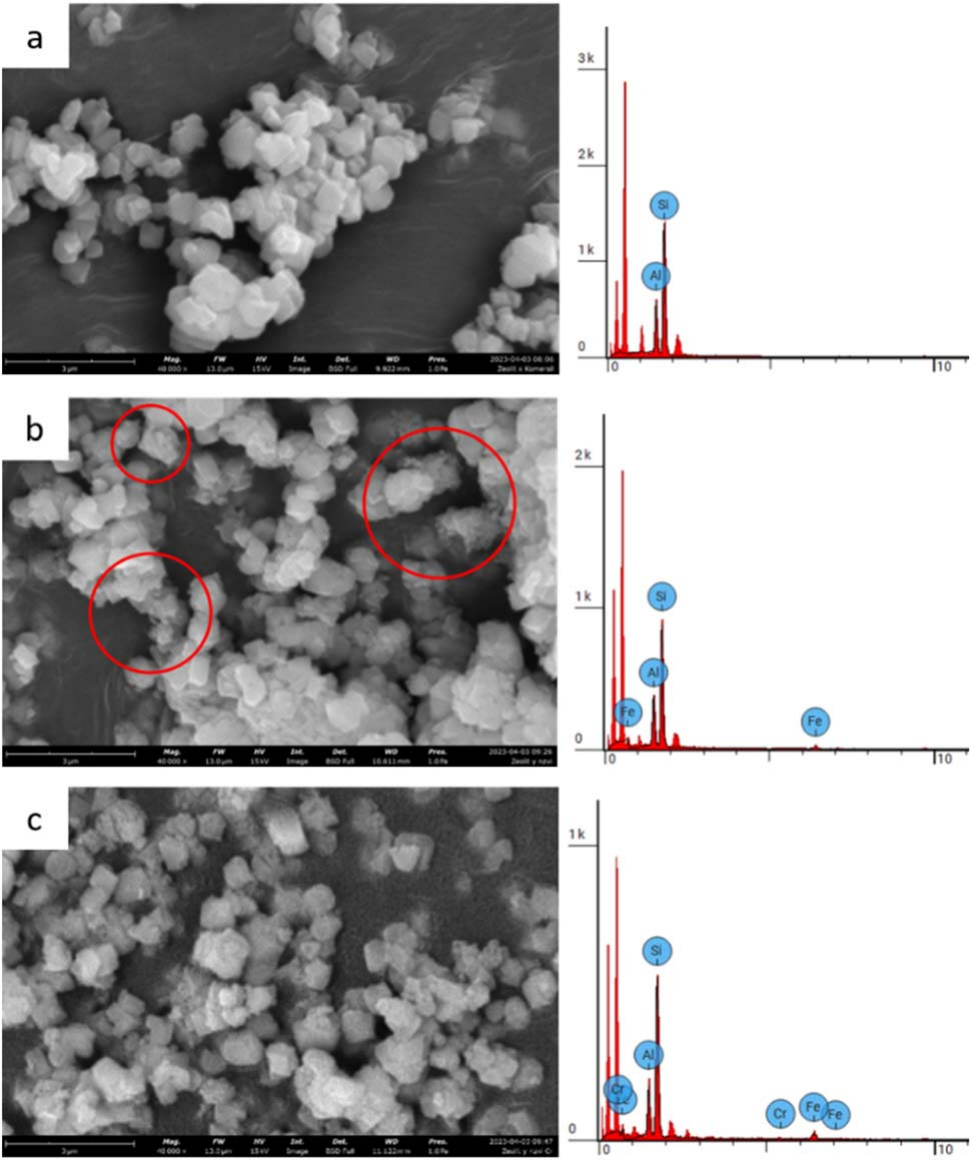
SEM-EDX images of (a) zeolite Y, (b) zeolite Y/nZVI before adsorption, and (c) zeolite Y/nZVI after adsorption.

The morphology and elemental composition (Fe and Cr) of zeolite Y and zeolite Y/nZVI before and after adsorption were analyzed using SEM–EDX. Zeolite Y particles exhibited a hexagonal crystalline form with a polished surface, whereas zeolite Y/nZVI tended to have a rough texture owing to the numerous nodules on its surface (Fig. 3a). These nodules were spherical Fe^0^ nanoparticles (nZVI) distributed without aggregation on the surface of zeolite Y (red circles, Fig. 3b).^35^

**Fig. 4:**
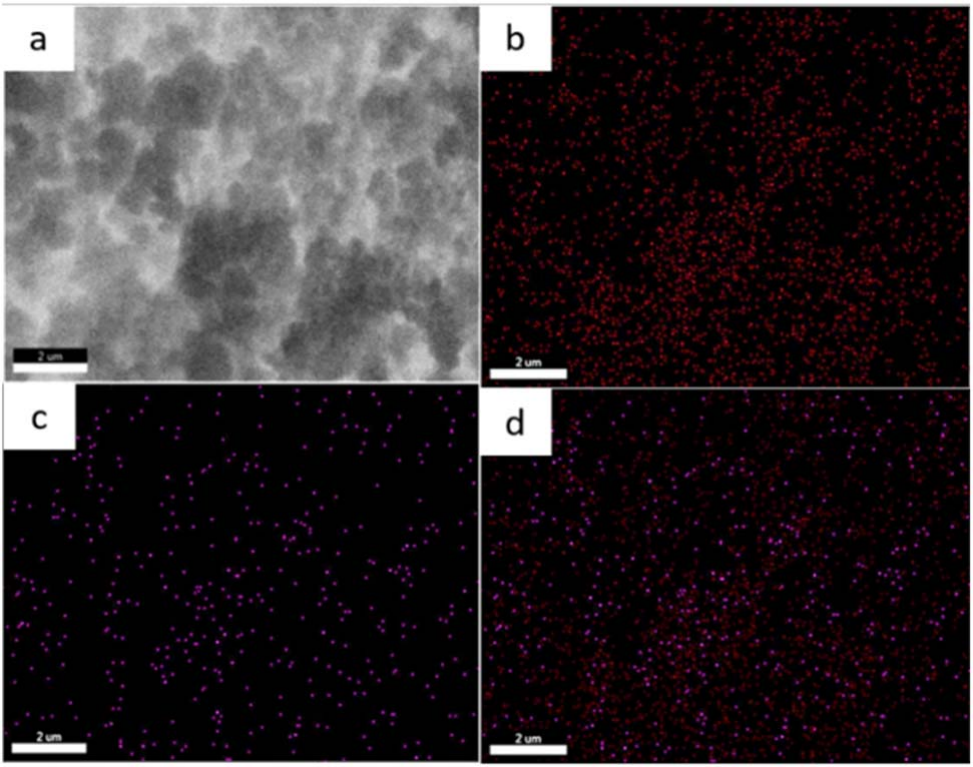
(a) SEM image of zeolite Y/nZVI-Cr, and SEM-EDX mapping of (b) Fe, (c) Cr, and (d) combined Fe and Cr analysis of zeolite Y/nZVI-Cr.

The EDX analysis of zeolite Y/nZVI indicated an Fe content of 10.47 wt% (Fig. 3c). The elemental mapping of zeolite Y/nZVI-Cr (Fig. 4) after adsorption shows that Cr is distributed in close proximity to Fe, indicating that reduced chromium species are associated with iron-rich regions on the adsorbent surface. This spatial correlation suggests the immobilization of Cr(iii) species following Cr(vi) reduction.

#### PSA

**Fig. 5:**
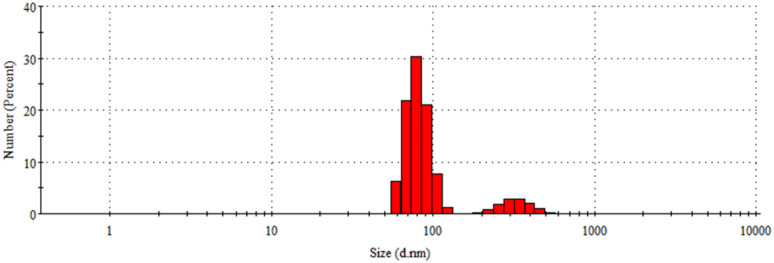
Particle size distribution of nZVI.

A particle size analyzer was used to determine the particle size distribution of Fe^0^ (nZVI). Fig. 5 shows that, based on numbers, 88.3% of nZVI has a particle size of 80.6 ± 10.8 nm, and 11.7% has a particle size 330.0 ± 234.5 nm. In addition, the PSA results show that the nZVI polydispersity index is 0.562, indicating a heterogeneous particle size distribution.^36^ The broad particle size distribution may influence adsorption efficiency by affecting the availability of active surface sites and mass transfer behavior.

#### pH_pzc_ analysis

**Fig. 6:**
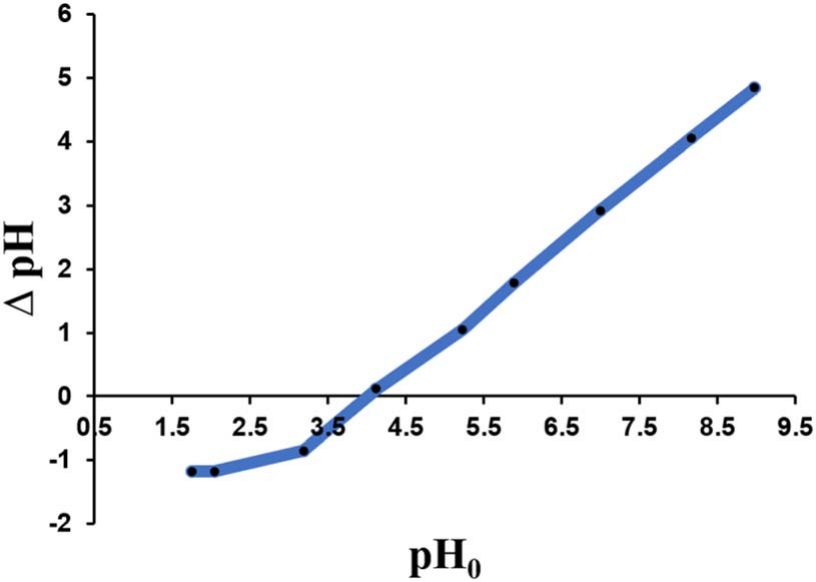
pH_pzc_ of zeolite Y/nZVI.

pH_pzc_ is the pH at which the negative and positive charges on the surface of the adsorbent are balanced, indicating that the overall surface charge is zero. At pH < pH_pzc_, H^+^ ions are generally found on the surface of the adsorbent, resulting in a positively charged surface. By contrast, when pH > pH_pzc_, H^+^ ions are more stable in the solution, causing the surface of the adsorbent to acquire a negative charge.^23,37^Fig. 6 shows that the pH_PZC_ value of zeolite Y/nZVI was 4, indicating that the surface of zeolite Y/nZVI carries a positive charge in the pH range of 1–4, which facilitates electrostatic interactions with the anionic species Cr(vi).

### Cr(vi) adsorption performance test

#### Effect of pH

**Fig. 7:**
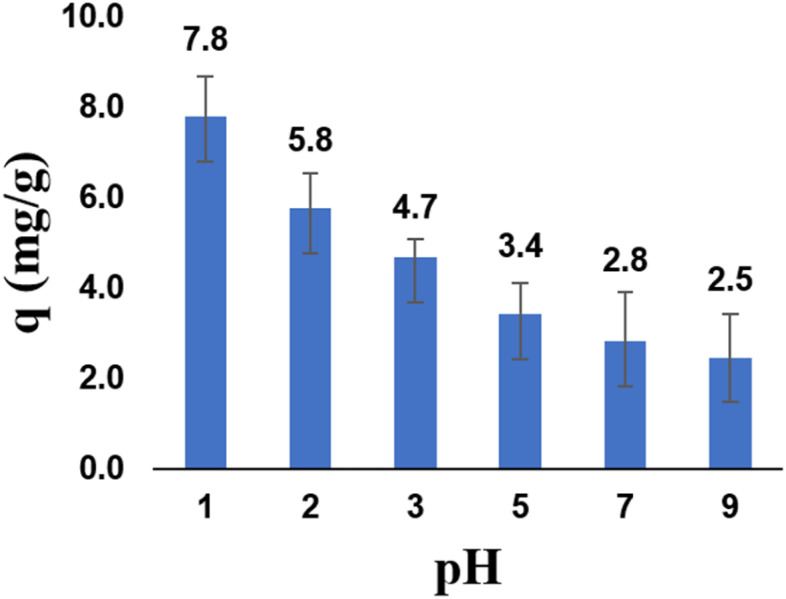
Effect of pH on zeolite Y/nZVI adsorption capacity.

The effect of solution pH on Cr(vi) adsorption by zeolite Y/nZVI is shown in Fig. 7. The adsorption performance is highly sensitive to the pH of the aqueous medium. A further decrease in the solution pH led to an increase in Cr(vi) adsorption. The optimal adsorption of Cr(vi) was achieved at a pH of 1 (acidic medium). This happens because the Cr(vi) reduction process requires not only electrons from nZVI but also protons (H^+^) at the solution/adsorbent surface.^38^ The reduction of Cr(vi) by nZVI can be represented by the following equation:^39^2HCrO_4_^−^ + 3Fe^0^ + 14H^+^ → 3Fe^2+^ + 2Cr^3+^ + 8H_2_O2CrO_4_^2−^ + 3Fe^0^ + 16H^+^ → 3Fe^2+^ + 2Cr^3+^ + 8H_2_OHCrO_4_^−^ + 3Fe^2+^ + 7H^+^ → 3Fe^3+^ + Cr^3+^ + 4H_2_OCrO_4_^2−^ + 3Fe^2+^ + 7H^+^ → 3Fe^3+^ + Cr^3+^ + 4H_2_O

According to Saleh *et al.*, Cr(vi) predominantly exists as hydrogen chromate (HCrO_4_^−^) in the pH range of 1–6, which is negatively charged.^17^ Under acidic conditions, the adsorbent surface becomes protonated, resulting in electrostatic attraction between the anionic Cr(vi) species and the positively charged surface.^40^ This mechanism is consistent with the measured pH_pzc_ value of 4 for zeolite Y/nZVI. The adsorbent surface carries a positive charge when the solution pH is below pH_pzc_. Therefore, at pH 1, the surface of zeolite Y/nZVI was positively charged, favoring the adsorption of anionic Cr(vi) species.

According to Cai *et al.*, when the pH of the solution is high, all hydroxide ions (OH^−^) react to form a layer of Fe(iii)/Cr(iii) hydroxide.^33^ The passivation layer obstructs the interaction between anionic species of Cr(vi) and the active site on the adsorbent surface, causing low Cr(vi) adsorption efficiency at high pH. Based on these results, Cr(vi) adsorption reaches 7.79 mg g^−1^ at pH 1.

#### Effect of temperature

**Fig. 8:**
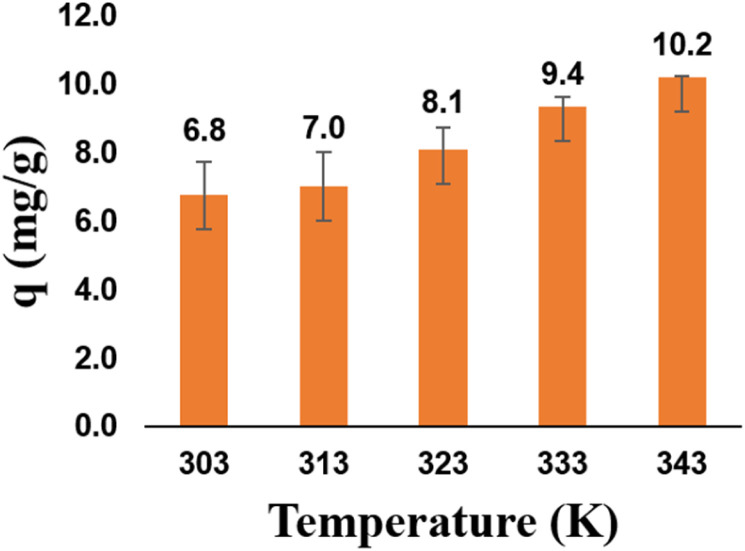
Effect of temperature on Cr(vi) reduction.

The molecular interaction between heavy metals and the adsorbent is strongly influenced by temperature.^41^ As shown in Fig. 8, the adsorption capacity of zeolite Y/nZVI increases with increasing temperature within the range of 303–343 K. This can be explained by the fact that higher temperatures can enhance the number of active sites on the adsorbent and promote the diffusion and mobility of molecules from the liquid phase to the adsorbent surface.^42,43^ With increasing temperature, the increasing adsorption capacity of zeolite Y/nZVI indicates that the adsorption process is endothermic. We can infer from these findings that at 343 K, the highest adsorption capacity of zeolite Y/nZVI for Cr(vi) is achieved (10.22 mg g^−1^).

#### Effect of Cr(vi) initial concentration

**Fig. 9:**
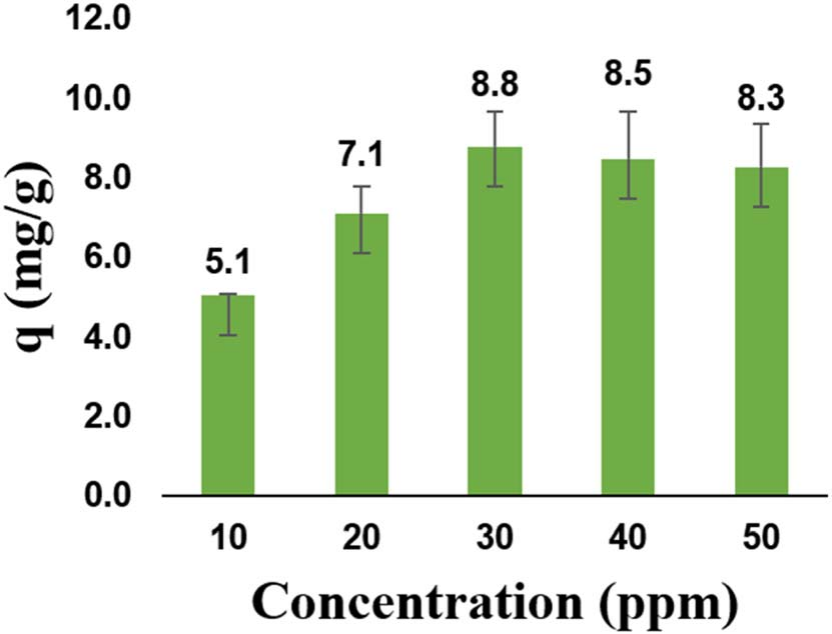
Effect of initial concentration on Cr(vi) reduction.

Fig. 9 shows that the adsorption capacity increases as the Cr(vi) concentration increases. However, when the Cr(vi) concentration exceeded 30 ppm, the adsorption capacity decreased. This reduction can be attributed to the limited number of active sites available for the zeolite Y/nZVI adsorbent. At higher initial concentrations, the mass transfer rate of Cr(vi) to the zeolite Y/nZVI surface increased. Once the surface became saturated with Cr(vi), further interactions between Cr(vi) and zeolite Y/nZVI were impeded.^1^ These results indicate that at a Cr(vi) concentration of 30 ppm, the adsorption process attained a maximum capacity of 8.80 mg g^−1^.

#### Effect of contact time

**Fig. 10:**
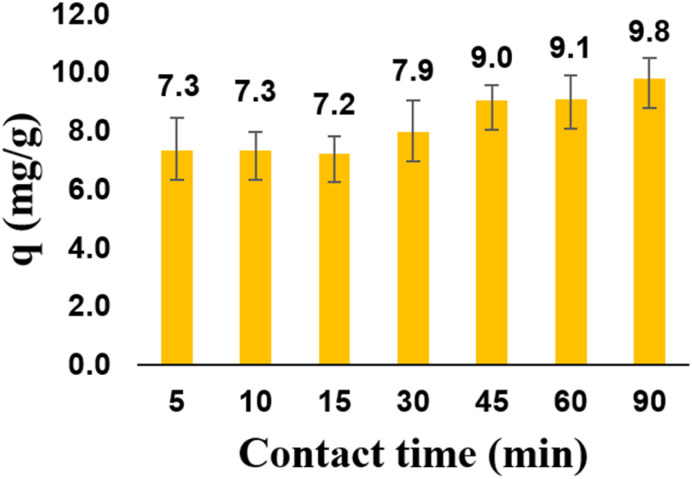
Effect of contact time on zeolite Y/nZVI adsorption capacity.

As shown in Fig. 10, the adsorption capacity of zeolite Y/nZVI increased with contact time (5–45 min). However, after 45 min, the adsorption capacity did not significantly increase. This is because at the initial contact time, active sites on the adsorbent are still available (vacant) in large numbers. At a contact time of 45 min, equilibrium was reached, such that Cr(vi) was no longer adsorbed as time progressed. At this equilibrium time, the desorption rate of Cr(vi) from the surface of zeolite Y/nZVI is equal to its adsorption rate, and both processes remain in a state of dynamic equilibrium.^1^ Based on these results, at a contact time of 45 min, the Cr(vi) adsorption process reaches an optimum capacity of 9.03 mg g^−1^.

### Isotherm adsorption

**Fig. 11:**
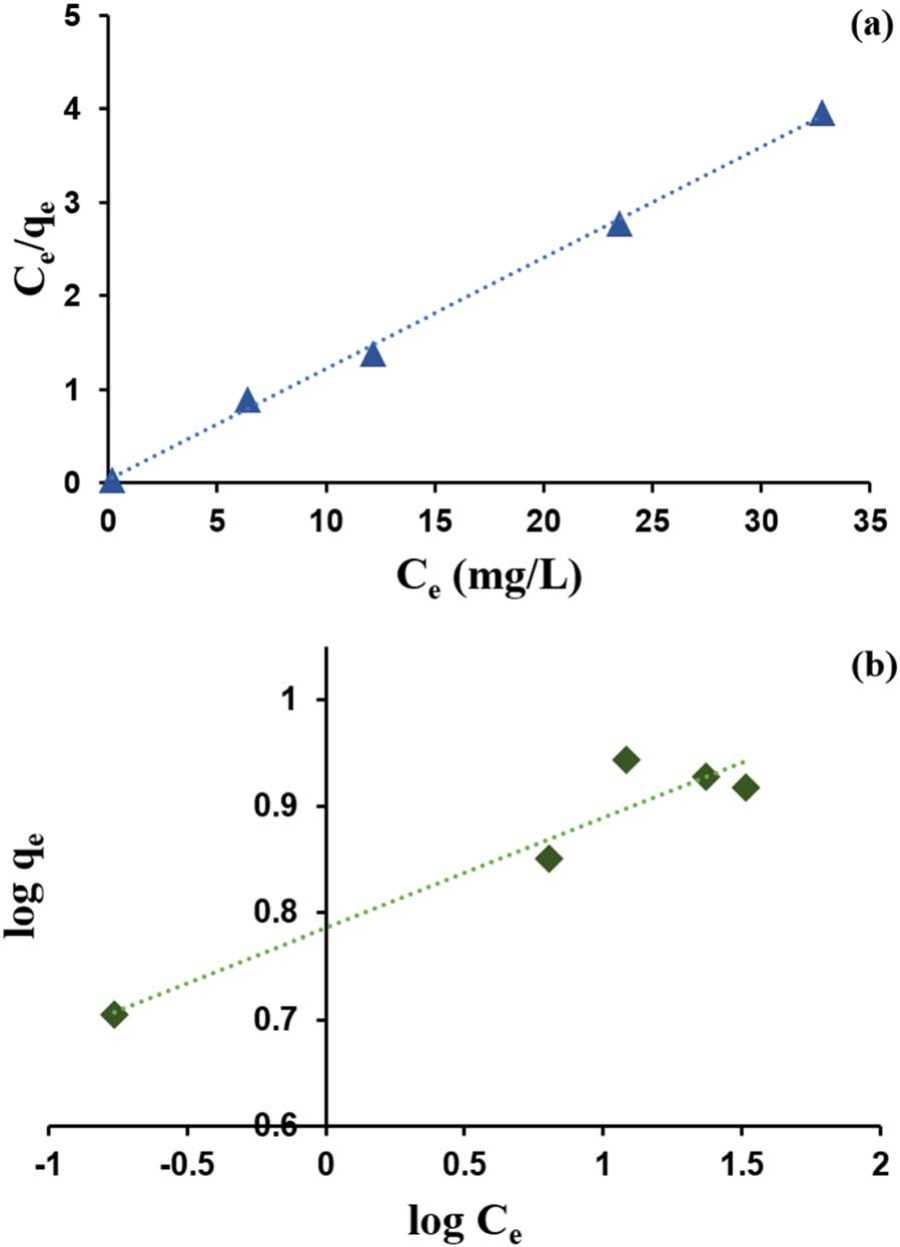
Adsorption isotherm curves of zeolite Y/nZVI model: (a) Langmuir and (b) Freundlich.

An isotherm study was conducted to quantitatively describe the interaction between Cr(vi) and zeolite Y/nZVI, and to understand the adsorption mechanism.^44^ Langmuir and Freundlich isotherm models were used. The calculation results for each isotherm parameter are presented in Table 1 and Fig. 11.

**Table 1:** Langmuir and Freundlich adsorption isotherm parameters

Langmuir		Freundlich	
*q*_max_ (mg g^−1^)	8.432	*K*_f_ (L g^−1^)	6.1136
*K*_L_ (L mg^−1^)	3.753	1/*n*	0.103
*R*_L_	0.025	*n*	9.689
*R* ^2^	0.9974	*R* ^2^	0.9203

As shown in Table 1, using the Langmuir isotherm model, zeolite Y/nZVI exhibited a maximum adsorption capacity (*q*_max_) of 8.432 mg g^−1^. The large Langmuir constant (*K*_L_) (3.753 L mg^−1^) indicates a strong interaction between zeolite Y/nZVI and Cr(vi). A separation factor of 0.025 indicates that 0 < RL < 1, indicating that the adsorption system was favorable.^1^

**Fig. 12:**
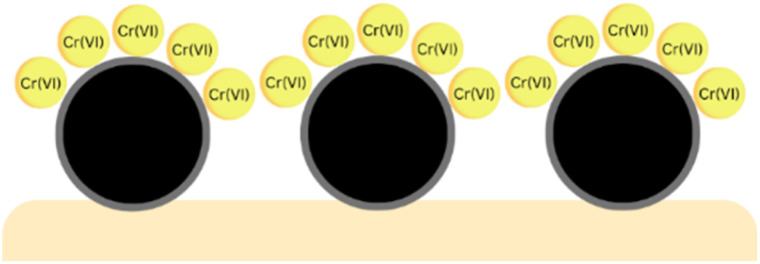
Illustration of single-layer formation during the adsorption process.

Fig. 11 shows that the correlation coefficient of the Langmuir isotherm is closer to 1 than that of the Freundlich isotherm. This indicates that Cr(vi) adsorption by zeolite Y/nZVI followed the Langmuir isotherm model. The Langmuir isotherm model indicated that Cr(vi) adsorption occurred *via* the formation of a homogeneous monolayer on the adsorbent surface (Fig. 12). In addition, the adsorbent possesses a limited number of active (vacant) sites that interact with the adsorbate, and significant interactions occur between the adsorbed species owing to steric hindrance on the surface.^1^

Although the Langmuir model better fits Cr(vi) adsorption onto zeolite Y/nZVI, Table 1 indicates that the Freundlich isotherm also yields a reasonably good correlation (*R*^2^ = 0.9203), suggesting that the zeolite Y/nZVI surface possesses a certain degree of heterogeneity.^45^ This suggests that, while monolayer adsorption on relatively uniform sites is the dominant mechanism, heterogeneous surface interactions also contribute to the overall Cr(vi) removal by zeolite Y/nZVI.

### Adsorption kinetics

**Fig. 13:**
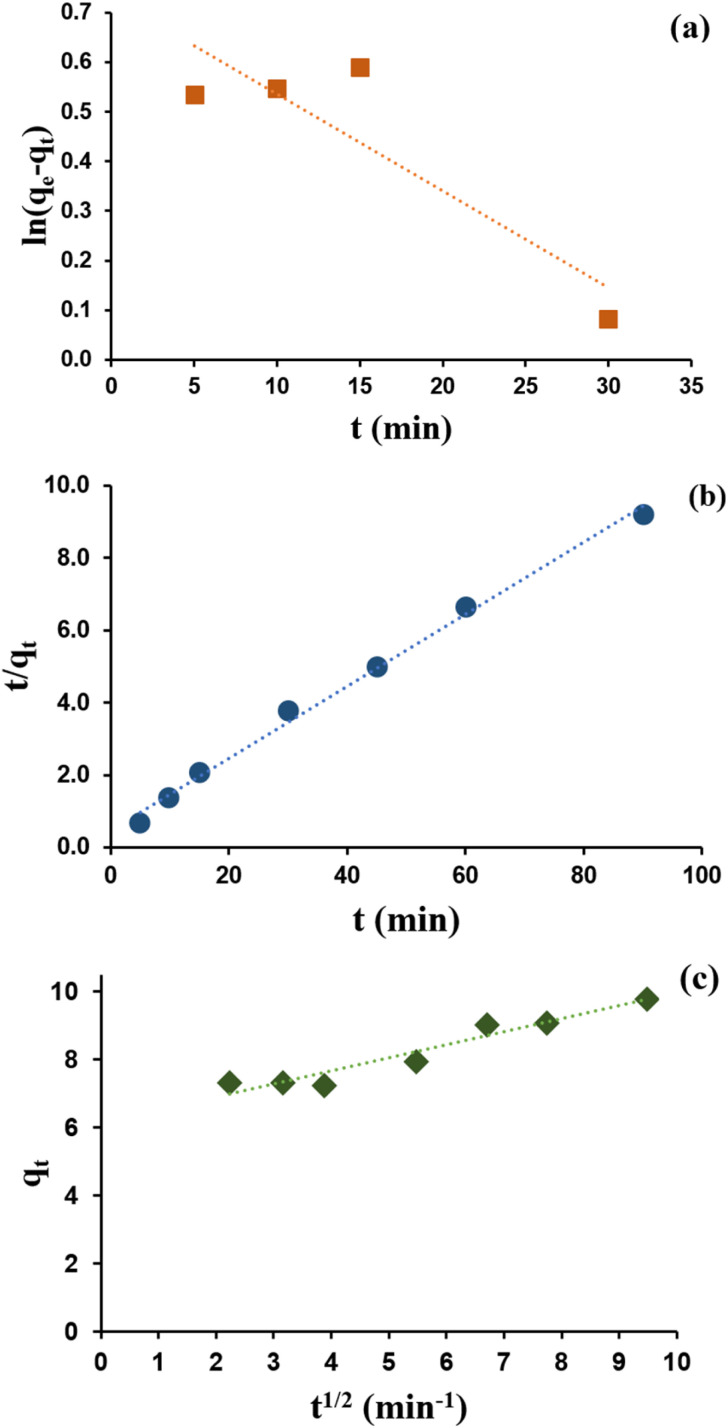
Adsorption kinetics curve of zeolite Y/nZVI model: (a) pseudo-first-order, (b) pseudo-second-order, and (c) intra-particle diffusion.

Kinetic studies were conducted to determine the adsorption rate and rate-determining steps.^1^ Pseudo-first-order and pseudo-second-order adsorption reaction kinetic models were used along with the adsorption–diffusion kinetics model as the intra-particle diffusion model. The curves and calculation results for each kinetic model parameter are presented in Fig. 13 and Table 2, respectively.

Adsorption kinetic parameters for pseudo-first-order, pseudo-second-order, and intra-particle diffusion models

**Table:** 

No.	Model	*R* ^2^	*k*	*q*_e_ (mg g^−1^)
1	Pseudo first order	0.7839	0.0196	2.08
2	Pseudo second order	0.9949	0.0206	10.06

**Table:** 

No.	Model	*R* ^2^	*R*_i_	Zone
3	Intra-particle diffusion	0.9303	0.3747	3

Table 2 shows that the correlation coefficient of the pseudo-second-order kinetics curve (*R*^2^ = 0.9949) is closer to 1 than the pseudo-first-order kinetics curve (*R*^2^ = 0.7839). This reveals that the adsorption of Cr(vi) using zeolite Y/nZVI follows pseudo-second-order kinetics, with an adsorption rate constant (*k*) of 0.0206 g mg^−1^ min^−1^ and adsorption capacity at equilibrium (*q*_e_) of 10.06 mg g^−1^. Based on the assumption of the pseudo-second-order kinetic model, the rate-determining step in the adsorption of Cr(vi) metal is chemical adsorption (chemisorption), and the adsorption capacity is highly dependent on the active sites on zeolite Y/nZVI.^1^

As shown in Fig. 13c, the plot of *q*_*t*_*vs. t*^1/2^ does not pass through (0,0), indicating that intra-particle diffusion is not the only step controlling the adsorption rate.^46^ Furthermore, the *R*_i_ value (0.3747, Table 2), which lies within the range of 0.1 < *R*_i_ < 0.5 (zone 3, strongly initial adsorption), indicates that intra-particle diffusion plays a significant role but is not the sole rate-limiting step.^47^ This confirms that Cr(vi) uptake by zeolite Y/nZVI proceeds through multiple mass transfer stages. The moderate *R*_i_ value also suggests that boundary layer diffusion contributes to mass transfer resistance but does not strongly limit the overall process. This behavior may be attributed to continuous agitation during the adsorption experiments, which reduced the thickness of the liquid film surrounding the adsorbent particles, as well as to the high surface reactivity of zeolite Y/nZVI. The rapid adsorption and reduction of Cr(vi) enhanced the concentration gradient between the bulk solution and the adsorbent surface, facilitating mass transfer.

### Thermodynamics

**Fig. 14:**
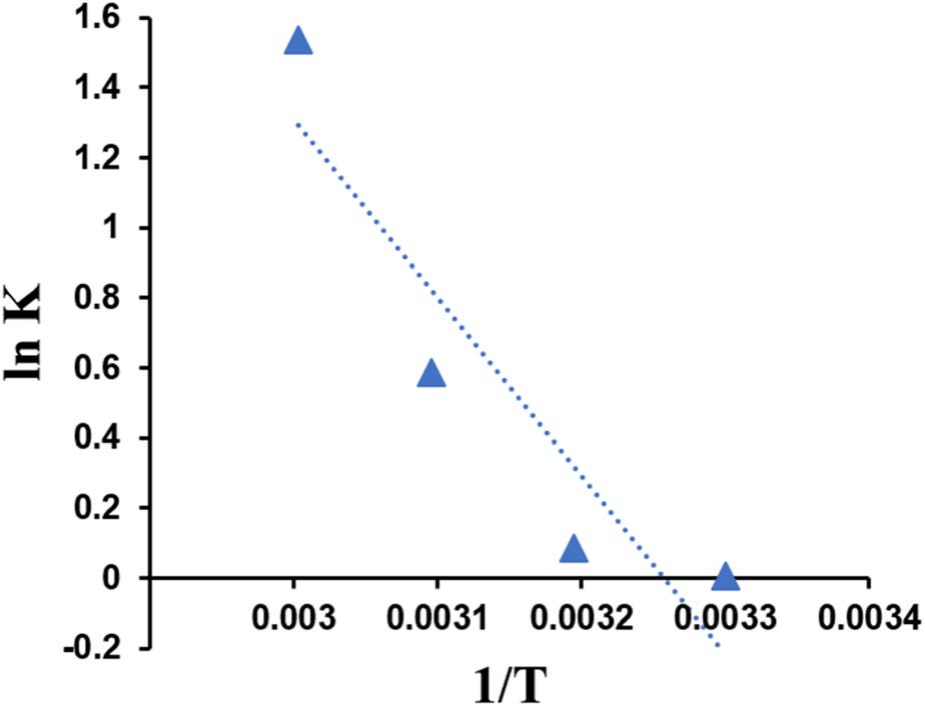
Van't Hoff adsorption curve of zeolite Y/nZVI.

The effect of temperature on the adsorption of Cr(vi) by zeolite Y/nZVI was evaluated through the analysis of thermodynamic parameters, namely, the enthalpy change (Δ*H*°), entropy change (Δ*S*°), and Gibbs free energy change (Δ*G*°), at temperatures of 303, 313, 323, and 333 K. To determine these parameters, the first step was to calculate the thermodynamic equilibrium constant (*K*) at each temperature, which was used to determine the Δ*G*° value. A plot of ln *K vs.* 1/*T* was then constructed to obtain the values of Δ*H*° and Δ*S*°, as shown in Fig. 14.

Based on Table 3, a positive Δ*H*° value is obtained, which indicates that the adsorption of Cr(vi) by zeolite Y/nZVI is endothermic. This can be explained by the increase in adsorption capacity with increasing temperature. In addition, enthalpy changes (Δ*H*°) in the 40–120 kJ mol^−1^ range indicate whether the interaction between Cr(vi) and zeolite Y/nZVI during adsorption occurs through chemical bonding or chemisorption. Meanwhile, a positive Δ*S*° value indicates high irregularity on the adsorbent surface and adsorbate during Cr(vi) adsorption by zeolite Y/nZVI. Based on Table 4, negative Δ*G*° values in the temperature range of 313–333 K indicate that adsorption is spontaneous at high temperatures.^48^ Although the Δ*G*° value at 303 K is slightly positive, this does not prevent Cr(vi) removal under practical conditions, as the mechanism involves coupled adsorption and reduction rather than simple reversible adsorption. The continuous reduction of Cr(vi) to Cr(iii), followed by immobilization through surface complexation and/or precipitation, shifts the reaction equilibrium and facilitates effective removal, even at near-ambient temperatures.

**Table 3:** Thermodynamic parameters of adsorption on zeolite Y/nZVI

Linear equation	Δ*H*° (kJ mol^−1^)	Δ*S*° (kJ mol^−1^)
*Y* = −5087*x* + 16.57	42.49	0.1377

**Table 4:** Δ*G*° values at 303, 313, 323, and 333 K

No	*T* (*K*)	Δ*G*° (kJ mol^−1^)
1	303	0.5511
2	313	−0.8265
3	323	−2.2041
4	333	−3.5818

### Comparison of Cr(vi) adsorption by zeolite Y/nZVI and other adsorbents

**Fig. 15:**
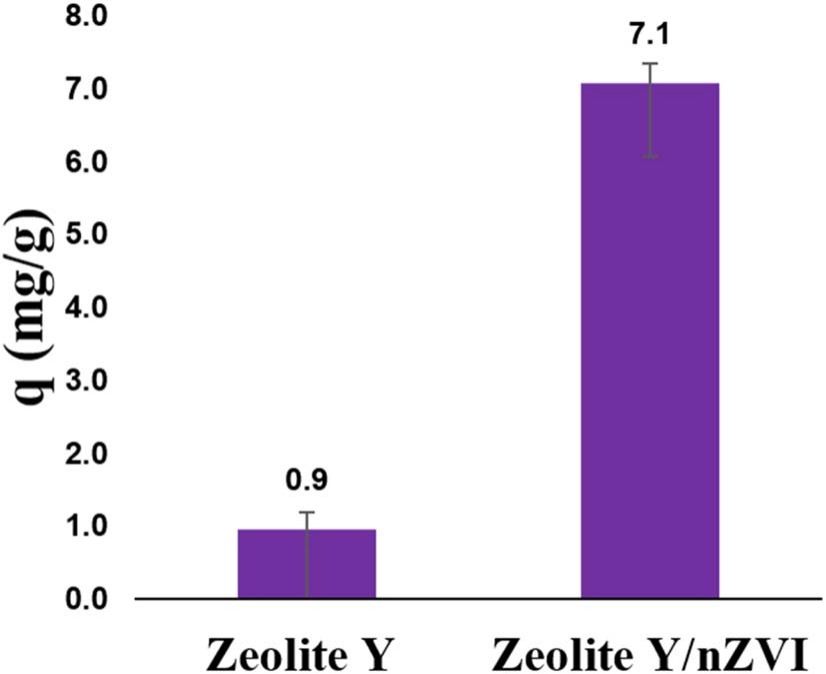
Comparison of Cr(vi) adsorption capacities between zeolite Y and zeolite Y/nZVI.

A comparison of the Cr(vi) adsorption capacities of zeolite Y and zeolite Y/nZVI is shown in Fig. 15. In this study, adsorption experiments were conducted under the optimum pH, concentration, and contact time conditions. As shown in Fig. 15, the Cr(vi) adsorption capacity of zeolite Y/nZVI was 7.4-fold higher than that of zeolite Y. This indicates that the modification of zeolite Y with nZVI significantly enhanced its adsorption capacity toward Cr(vi).

A comparison of the Cr(vi) removal capacities of various adsorbents is summarized in Table 5. The green-synthesized zero-valent iron (Fe^0^) cannot be readily compared with other reported adsorbents owing to differences in experimental conditions, such as the initial Cr(vi) concentration, pH, contact time, and adsorbent dosage. These parameters strongly influence the reported adsorption capacity and removal efficiency. Therefore, the values presented in Table 5 are intended to provide a general performance overview, rather than a direct comparison with other adsorbents. Despite its moderate adsorption capacity, the zeolite Y/nZVI composite developed in this study demonstrated a competitive performance, considering its simple green synthesis route and the combined adsorption, reduction, and precipitation mechanisms involved in Cr(vi) removal. Furthermore, it can be applied directly in the field.

**Table 5:** Cr(vi) removal capacities for various adsorbents

Adsorbent	Adsorption capacity (mg g^−1^)	References
Green synthesis zeolite Y/nZVI	7.1	In this work
Zeolites modified ZVI	2.49	49
Green synthesis Fe NPs	14.3	27
W-nZVI	35	50
Green low-cost synthesis P-NZVI	44.47	51

## Conclusions

The modification of zeolite Y with nZVI enhanced its performance in reducing Cr(vi) concentration. The adsorption capacity of zeolite Y increased from 0.9 mg g^−1^ to 7.1 mg g^−1^. The temperature and pH strongly influenced the adsorption of Cr(vi) by zeolite Y/nZVI. A temperature increase improved the adsorption capacity, with the highest value of 10.22 mg g^−1^ observed at 343 K. Cr(vi) adsorption by zeolite Y/nZVI was optimal under acidic conditions, particularly at pH 1. Based on the results obtained, zeolite Y/nZVI has the potential to be applied on a larger scale for the reduction of Cr(vi) levels.

## Conflicts of interest

The authors declare no conflicts of interest.

## Supplementary Material

RA-016-D5RA09655C-s001

## Data Availability

The data supporting this article have been included as part of the supplementary information (SI). Dataset available at Dryad repository at https://doi.org/10.5061/dryad.sbcc2frnt. Supplementary information is available. See DOI: https://doi.org/10.1039/d5ra09655c.
