# Supplementary material for: Green-synthesized zeolite Y-supported zero-valent iron nanocomposite for enhanced adsorptive reduction of hexavalent chromium from aqueous solutions
Source: RSC Adv. 2026 May 15;16(28):26155–64. doi: 10.1039/d5ra09655c (PMC13178597; doi:10.1039/d5ra09655c)
Supplement: RA-016-D5RA09655C-s001 [file RA-016-D5RA09655C-s001.pdf]

Supplementary Information (SI) for RSC Advances.
This journal is © The Royal Society of Chemistry 2026

Supporting Information

Green-Synthesized Zeolite Y–Supported Zero-Valent Iron Nanocomposite for Enhanced Adsorptive Reduction of Hexavalent Chromium from Aqueous Solutions

Nur Fariha Mahmuda,[a] Yanuardi Raharjo,[a,b,*] Tokok Adiarto,[a,b] Handoko Darmokoesoemo,[b] Heru Pramono[c] and Ahmad Fauzi Ismail [a,d]

a. Composite Materials & Applications Research Group (MSTRG), Chemistry Department, Faculty of Science and Technology, Universitas Airlangga, Surabaya 60115, Indonesia

b. Chemistry Department, Faculty of Science and Technology Universitas Airlangga, Surabaya 60115, Indonesia

c. Laboratory of Fisheries Microbiology, Department of Marine Science, Faculty of Fisheries and Marine, Universitas Airlangga, Surabaya 60115, Indonesia

d. Advances Membrane Technology Research Centre (AMTEC), Universiti Teknologi Malaysia, Skudai, 81310, Malaysia

1. Synthesis of Zeolite Y/nZVI

Step 1: Zeolite Y (4 g) was added to 50 mL of 0.1 M $FeCl_3·6H_2O$ solution in a 250 mL Erlenmeyer flask (8% w/v) and magnetically stirred for 30 min.

Step 2: 50 mL of the green tea extract was added dropwise to the Zeolite Y mixture and continuously stirred for 30 min.

Step 3: The black suspension was centrifuged at 4000 rpm for 15 min to collect the residue.

Step 4: The residue was washed twice with DI water and twice with absolute ethanol.

Step 5: The black residue was then transferred to an evaporating dish and dried in a vacuum desiccator at room temperature (34°C). The powdered Zeolite Y/nZVI was stored in a vacuum desiccator until further use to prevent oxidation.

2. XRD of Zeolite Y

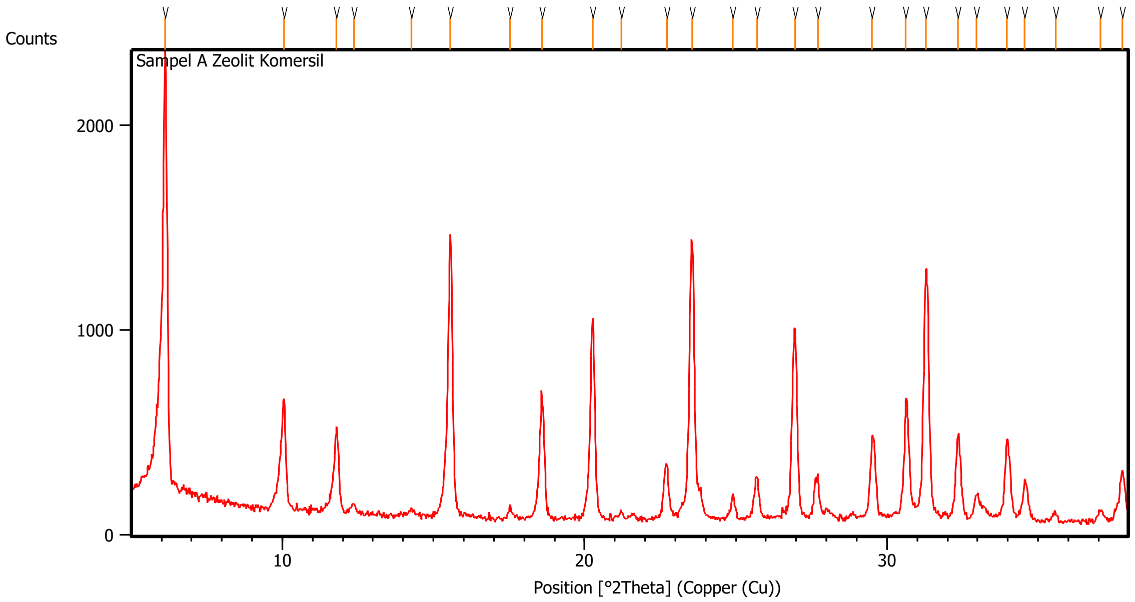

Peak List:

Pos. [°2Th.]	Height [cts]	FWHM Left [°2Th.]	d-spacing [Å]	Rel. Int. [%]
6.1302	2189.02	0.1338	14.41806	100.00
10.0556	539.13	0.1171	8.79674	24.63
11.7913	417.95	0.1338	7.50543	19.09
12.3862	41.79	0.1171	7.14627	1.91
14.2793	29.46	0.2007	6.20283	1.35
15.5603	1385.78	0.1506	5.69493	63.31
17.5224	53.41	0.1004	5.06142	2.44
18.5923	585.60	0.0836	4.77249	26.75
20.2630	987.90	0.1338	4.38262	45.13
21.2153	36.36	0.1004	4.18800	1.66
22.7197	265.24	0.1506	3.91398	12.12
23.5684	1339.72	0.1506	3.77492	61.20
24.9092	117.29	0.0669	3.57469	5.36
25.7024	207.50	0.1338	3.46614	9.48

26.9680	920.20	0.2007	3.30627	42.04
27.7075	223.63	0.1338	3.21969	10.22
29.5111	416.12	0.1171	3.02689	19.01
30.6280	592.68	0.1338	2.91901	27.08
31.2749	1190.55	0.1506	2.86010	54.39
32.3512	418.25	0.1506	2.76736	19.11
32.9613	126.67	0.1673	2.71752	5.79
33.9708	395.11	0.1171	2.63904	18.05
34.5579	203.26	0.0836	2.59554	9.29
35.5925	32.66	0.1673	2.52243	1.49
37.0728	50.24	0.2007	2.42504	2.30
37.7794	250.08	0.0836	2.38129	11.42

3. XRD of Zeolite Y/nZVI

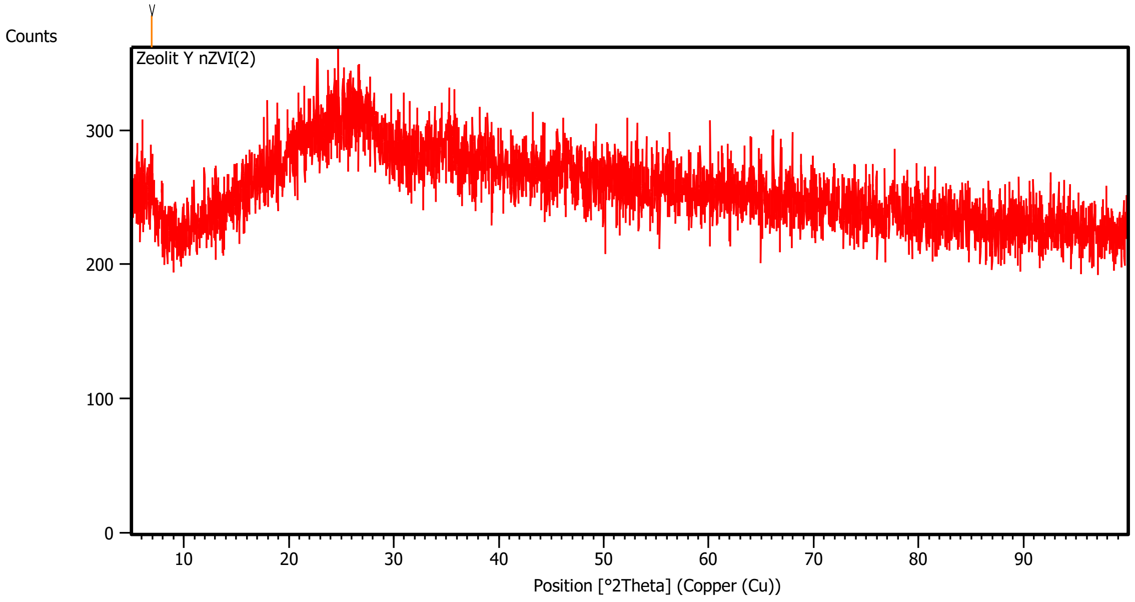

Peak List:

Pos. [°2Th.]	Height [cts]	FWHM Left [°2Th.]	d-spacing [Å]	Rel. Int. [%]
6.9201	19.48	0.9792	12.76329	100.00

4. Spectra FT-IR of Zeolite Y

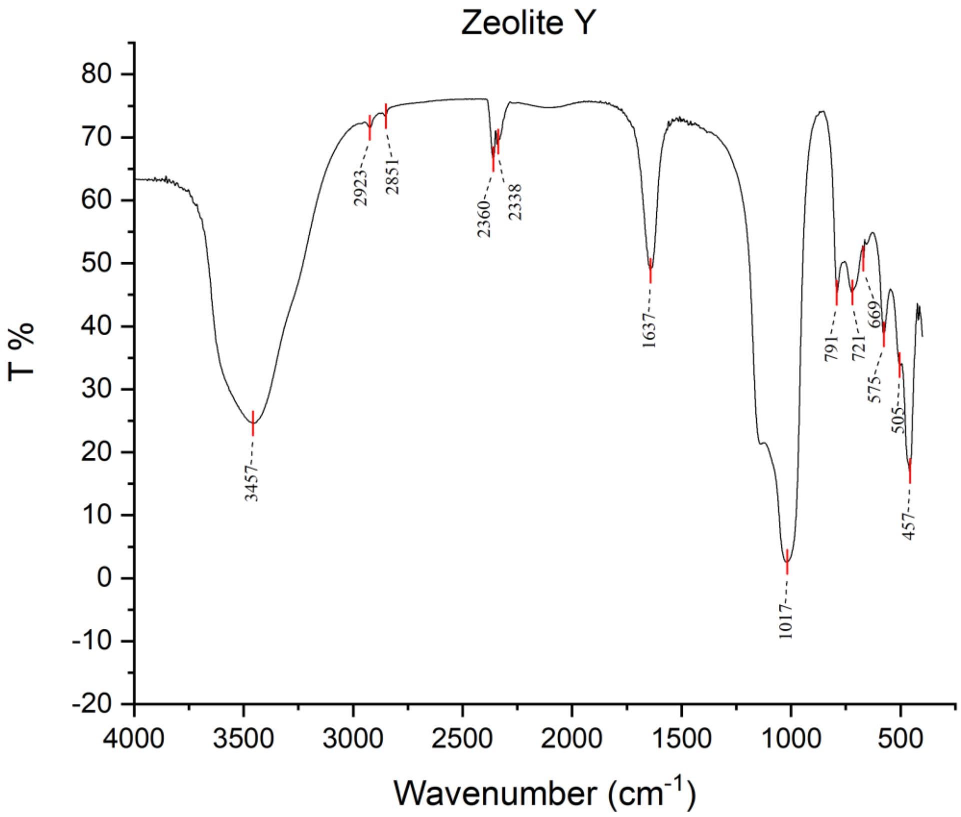

5. **Spectra FT-IR of Zeolite Y/nZVI**

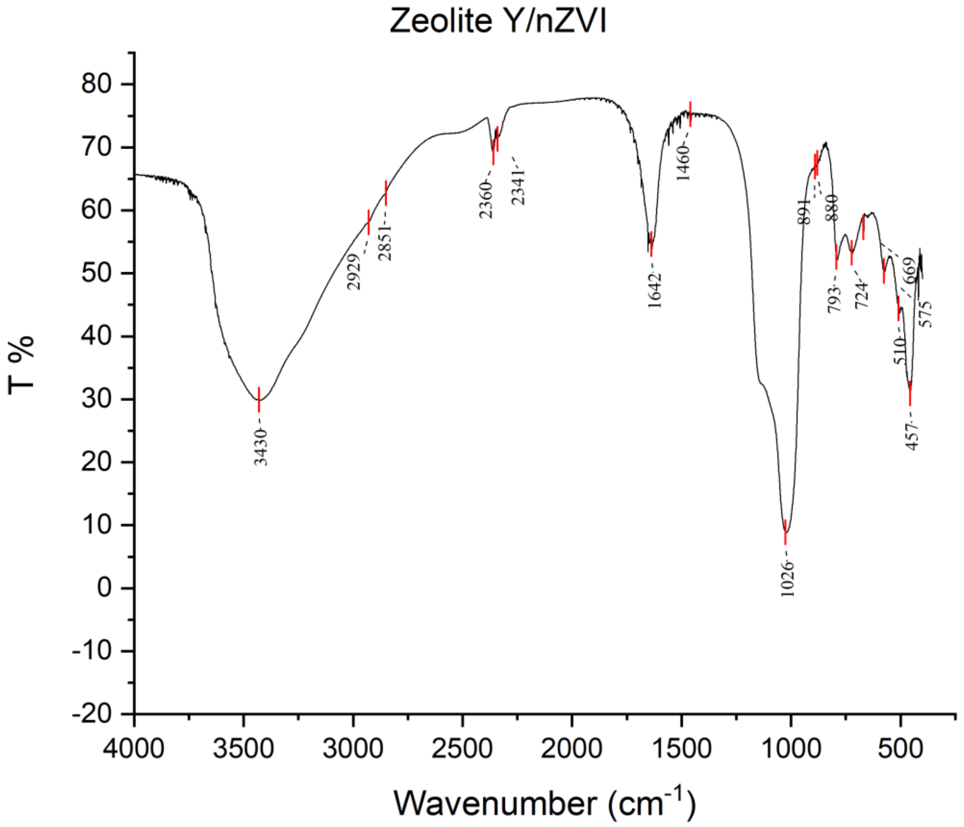

6. **Spectra FT-IR of Zeolite Y/nZVI-Cr**

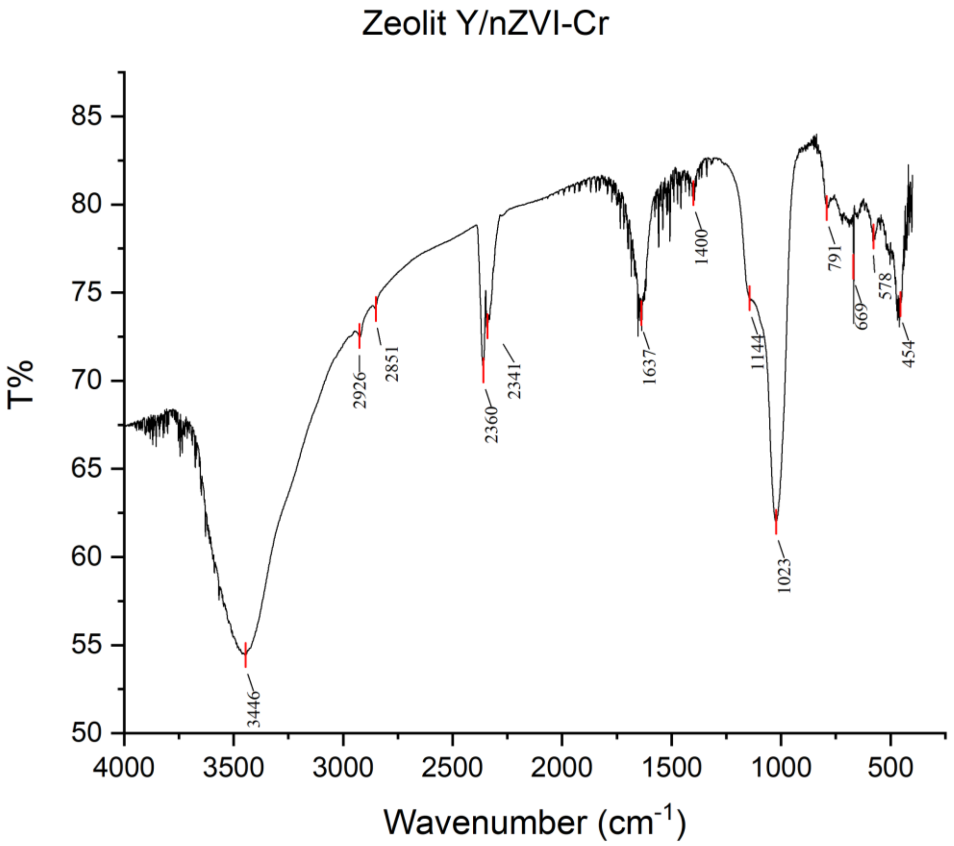

7. pH$_{PZC}$ of Zeolite Y/nZVI

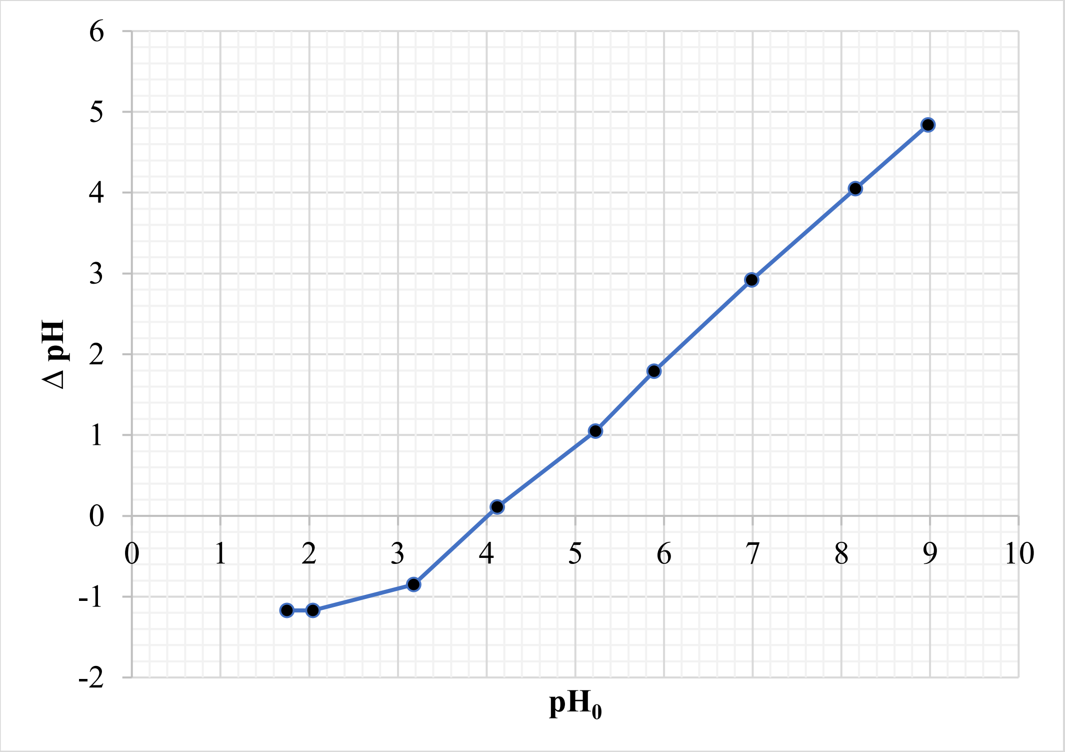

No	pH$_0$	Δ pH
1	1.75	-1.17
2	2.04	-1.17
3	3.18	-0.85
4	4.12	0.11
5	5.23	1.05
6	5.89	1.79
7	6.99	2.92
8	8.16	4.05
9	8.98	4.84
